# Supplementary material for: Agar Biopolymer Films for Biodegradable Packaging: A Reference Dataset for Exploring the Limits of Mechanical Performance
Source: Materials (Basel). 2022 Jun 1;15(11):3954. doi: 10.3390/ma15113954 (PMC9182270; doi:10.3390/ma15113954)
Supplement: Supplementary file 1 [file materials-15-03954-s001.zip › materials-1732196-supplementary.pdf]

materials

Article

Agar Biopolymer Films for Biodegradable Packaging: A reference dataset for exploring the limits of mechanical performance

Valentina Hernández[1], Davor Ibarra[2] , Johan F. Triana[3] , Bastian Martínez-Soto[4], Matías Faúndez[2], Diego A. Vasco[2], Leonardo Gordillo[3], Felipe Herrera[3,5], Claudio García-Herrera[2], , Alysia Garmulewicz [1,6]

1 Department of Administration, Faculty of Administration and Economics, University of Santiago of Chile (USACH), Avenida Libertador Bernardo O'Higgins 3363, Estación Central, Santiago 917022, Chile
2 Department of Mechanical Engineering, University of Santiago of Chile (USACH), Avenida Libertador Bernardo O'Higgins 3363, Santiago 917022, Chile
3 Department of Physics, University of Santiago of Chile (USACH), Avenida Victor Jara 3493, Santiago 9170124, Chile
4 Department of Mathematics and Computer Science, University of Santiago of Chile (USACH), Las Sophoras 173, Santiago 9170124, Chile
5 ANID-Millennium Institute for Research in Optics, Concepción 4030000, Chile
6 CABDyN Complexity Centre, University of Oxford, Oxford OX1 2JD, United Kingdom

* Correspondence: alysia.garmulewicz@usach.cl

1. ANOVA - Data range 1: Low ingredients concentrations:

In Equation (1) the Young modulus model in low ingredients concentrations

$$\text{Young Modulus [Mpa]} = -9.463 + (20.78 \times \%\text{Agar}) + (13.09 \times \%\text{Gly}) - (28.45 \times \%\text{Agar} \times \%\text{Gly}) \quad \text{(S1)}$$

Table S1. Statistic parameters for Young modulus model in low ingredients concentrations

Term	Effect	Coef	SE Coef	T-Value	P-Value	VIF
Constant		2.2548	0.0831	27.12	0.000	
% Agar	-3.7223	1.8612	0.0831	22.39	0.000	1.00
% Gly	-2.5777	-1.2888	0.0831	-15.50	0.000	1.00
% Agar × % Gly	-2.2223	-1.1112	0.0831	-13.36	0.000	1.00

Table S2. The correlation coefficient of the Young modulus model in low ingredients concentrations.

S	R-sq	R-sq(adj)	R-sq(pred)
0.371826	98.29%	97.97%	97.33%

Table S3. Analysis of variance of the Young modulus model in low ingredients concentrations.

Source	DF	Adj SS	Adj MS	F-Value	P-Value
Model	3	127.194	42.3981	306.67	0.000
Linear	2	102.501	51.2503	370.70	0.000
% Agar	1	69.279	69.2787	501.10	0.000
% Gly	1	33.222	33.2219	240.30	0.000

2-Way Interactions	1	24.694	24.6938	178.61	0.000
% Agar × % Gly	1	24.694	24.6938	178.61	0.000
Error	16	2.212	0.1383		
Total	19	129.406			

Table S4. Fits and Diagnostics for Unusual Observations for Young modulus model in low ingredients concentrations.

Obs	Young Modulus [Mpa]	Fit	Resid	Std Resid
11	5.350	6.516	-1.166	-3.51
13	7.240	6.516	0.724	2.18

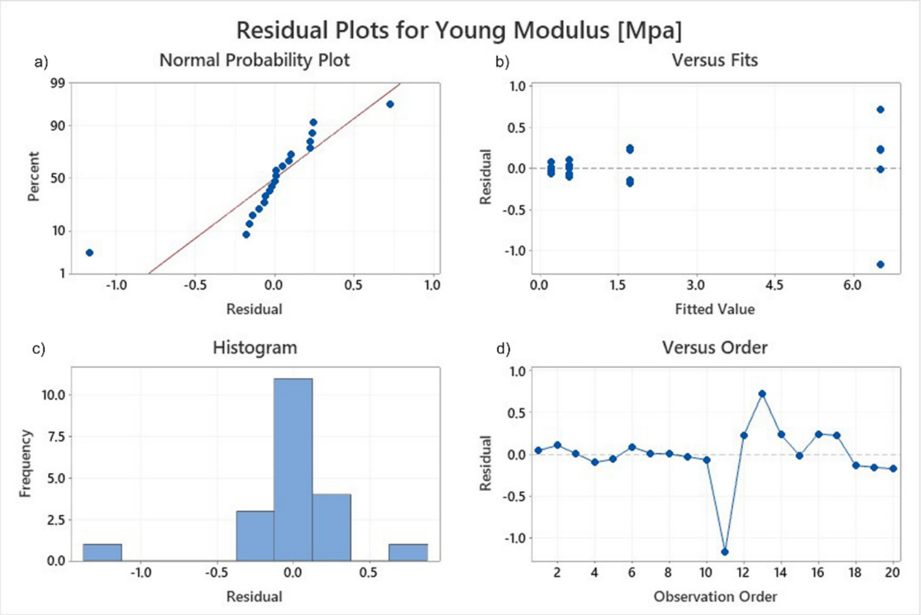

Figure S1. Analysis of residuals for Young modulus model in low ingredients concentrations. (**a**) Homoscedasticity analysis. (**b**) Residuals analysis. (**c**) Residuals distributions. (**d**) Randomized analysis.

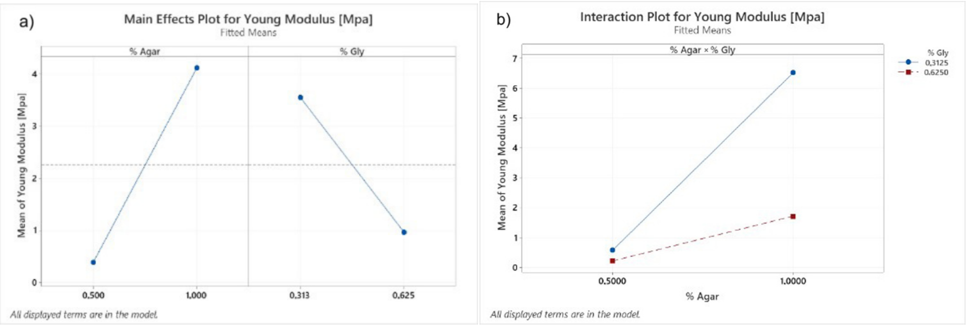

Figure S2. Effect analysis for Young modulus model in low ingredients concentrations. (**a**) Main effects. (**b**) Interaction effect.

In Equation (2) the elongation at break model in low ingredients concentrations

$$\text{Elongation at break [\%]} = 43.51 - (30.13 \times \%\text{Agar}) - (12.57 \times \%\text{Gly}) + (39.6 \times \%\text{Agar} \times \%\text{Gly}) \quad \text{(S2)}$$

Table S5. Statistic parameters for elongation at break model in low ingredients concentrations

Term	Effect	Coef	SE Coef	T-Value	P-Value	VIF
Constant		28.948	0.443	65.41	0.000	
% Agar	-5.778	-2.889	0.443	-6.53	0.000	1.00
% Gly	5.358	2.679	0.443	6.05	0.000	1.00
% Agar × % Gly	3.096	1.548	0.443	3.50	0.003	1.00

Table S6. The correlation coefficient of the elongation at break model in low ingredients concentrations.

S	R-sq	R-sq(adj)	R-sq(pred)
1.97925	85.11%	82.32%	76.74%

Table S7. Analysis of variance of the elongation at break model in low ingredients concentrations.

Source	DF	Adj SS	Adj MS	F-Value	*p*-Value
Model	3	358.34	119.447	30.49	0.000
Linear	2	310.43	155.215	39.62	0.000
% Agar	1	166.91	166.906	42.61	0.000
% Gly	1	143.52	143.524	36.64	0.000
2-Way Interactions	1	47.91	47.912	12.23	0.003
% Agar × % Gly	1	47.91	47.912	12.23	0.003
Error	16	62.68	3.917		
Total	3	358.34	119.447	30.49	0.000

Table S8. Fits and Diagnostics for Unusual Observations for elongation at break model in low ingredients concentrations.

Obs	Elongation at break [%]	Fit	Resid	Std Resid
6	28.950	32.968	-4.018	-2.27
16	26.275	30.286	-4.011	-2.27

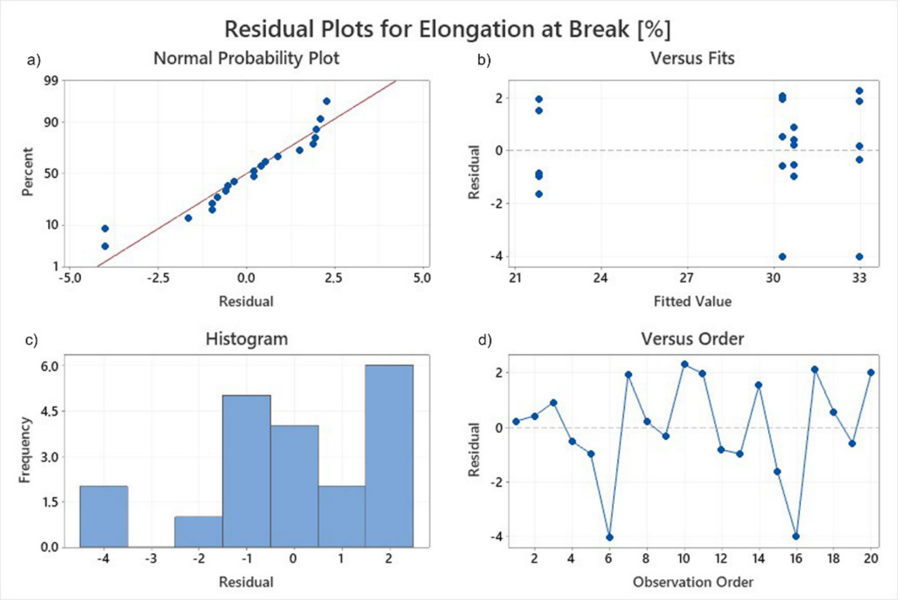

Figure S3. Analysis of residuals for elongation at break model in low ingredients concentrations. (**a**) Homoscedasticity analysis. (**b**) Residuals analysis. (**c**) Residuals distributions. (**d**) Randomized analysis.

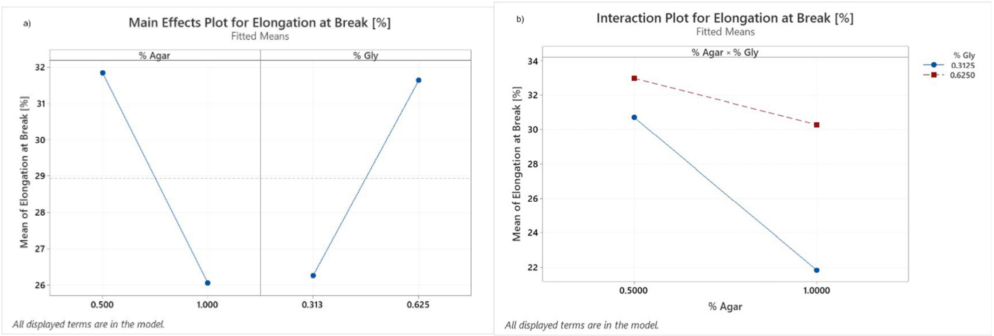

Figure S4. Effect analysis for elongation at break model in low ingredients concentrations. (**a**) Main effects. (**b**) Interaction effect.

In Equation (3) the Ultimate Tensile Strength (UTS) model in low ingredients concentrations

$$UTS\ [Mpa] = -21.44 + (57.04 \times \%Agar) + (25.06 \times \%Gly) - (65.38 \times \%Agar \times \%Gly) \quad (S3)$$

Table S9. Statistic parameters for UTS model in low ingredients concentrations

Term	Effect	Coef	SE Coef	T-Value	P-Value	VIF
Constant		10.103	0.216	46.72	0.000	
% Agar	13.198	6.599	0.216	30.52	0.000	1.00
% Gly	-7.491	-3.746	0.216	-17.32	0.000	1.00
% Agar × % Gly	-5.108	-2.554	0.216	-11.81	0.000	1.00

Table S10. The correlation coefficient of the UTS model in low ingredients concentrations.

S	R-sq	R-sq(adj)	R-sq(pred)
0.966992	98.85%	98.63%	98.20%

Table S11. Analysis of variance of the UTS model in low ingredients concentrations.

Source	DF	Adj SS	Adj MS	F-Value	P-Value
Model	3	1281.97	427.323	456.99	0.000
Linear	2	1151.53	575.767	615.74	0.000
% Agar	1	870.94	870.939	931.41	0.000
% Gly	1	280.59	280.595	300.08	0.000
2-Way Interactions	1	130.44	130.436	139.49	0.000
% Agar × % Gly	1	130.44	130.436	139.49	0.000
Error	16	14.96	0.935		
Total	19	1296.93			

Table S12. Fits and Diagnostics for Unusual Observations for UTS model in low ingredients concentrations.

Obs	UTS [Mpa]	Fit	Resid	Std Resid
17	12.252	10.402	1.849	2.14

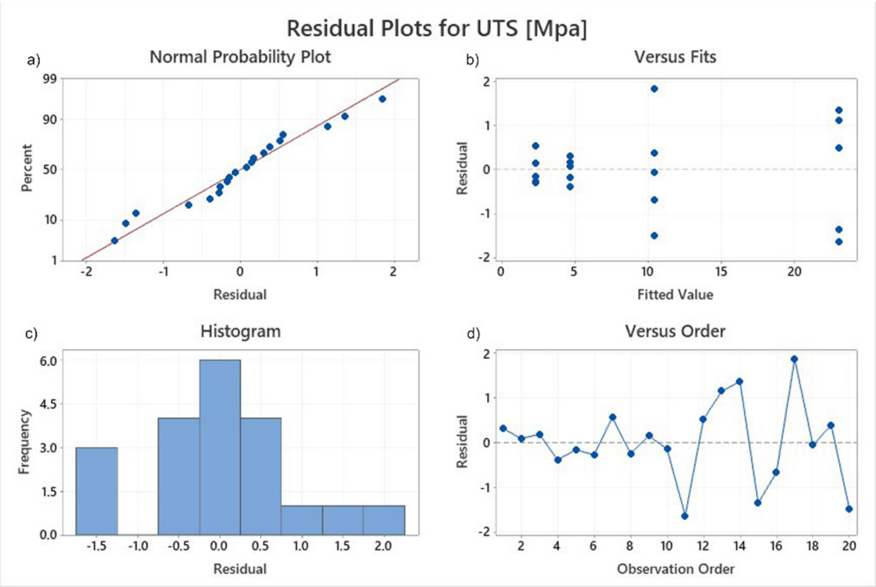

Figure S5. Analysis of residuals for UTS model in low ingredients concentrations. (**a**) Homoscedasticity analysis. (**b**) Residuals analysis. (**c**) Residuals distributions. (**d**) Randomized analysis.

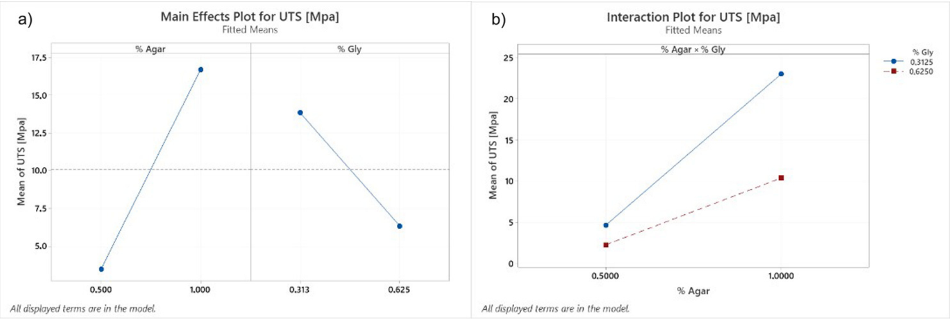

Figure S6. Effect analysis for UTS model in low ingredients concentrations. (**a**) Main effects. (**b**) Interaction effect.

2. ANOVA - Data range 2: Medium ingredients concentrations:

In Equation 4 the Young modulus model in medium ingredients concentrations

$$\text{Young Modulus [Mpa]} = 14.07 - (4.980 \times \%\text{Agar}) - (8.620 \times \%\text{Gly}) + (3.301 \times \%\text{Agar} \times \%\text{Gly}) \quad \text{(S4)}$$

Table S13. Statistic parameters for Young modulus model in medium ingredients concentrations

Term	Effect	Coef	SE Coef	T-Value	P-Value	VIF
Constant		1.0483	0.0422	24.85	0.000	
% Agar	0.1788	0.0894	0.0422	2.12	0.050	1.00
% Gly	-0.2289	-0.1145	0.0422	-2.71	0.015	1.00
% Agar × % Gly	1.0317	0.5158	0.0422	12.23	0.000	1.00

Table S14. The correlation coefficient of the Young modulus model in medium ingredients concentrations.

S	R-sq	R-sq(adj)	R-sq(pred)
0.188651	90.98%	89.29%	85.91%

Table S15. Analysis of variance of the Young modulus model in medium ingredients concentrations.

Source	DF	Adj SS	Adj MS	F-Value	P-Value
Model	3	5.7437	1.91456	53.80	0.000
Linear	2	0.4219	0.21097	5.93	0.012
% Agar	1	0.1599	0.15990	4.49	0.050
% Gly	1	0.2620	0.26204	7.36	0.015
2-Way Interactions	1	5.3218	5.32175	149.53	0.000
% Agar × % Gly	1	5.3218	5.32175	149.53	0.000
Error	16	0.5694	0.03559		
Total	19	6.3131			

Table S16. Fits and Diagnostics for Unusual Observations for Young modulus model in medium ingredients concentrations.

Obs	Young Modulus [Mpa]	Fit	Resid	Std Resid
12	0.3613	0.7364	-0.3750	-2.22
13	0.3633	0.7364	-0.3730	-2.21

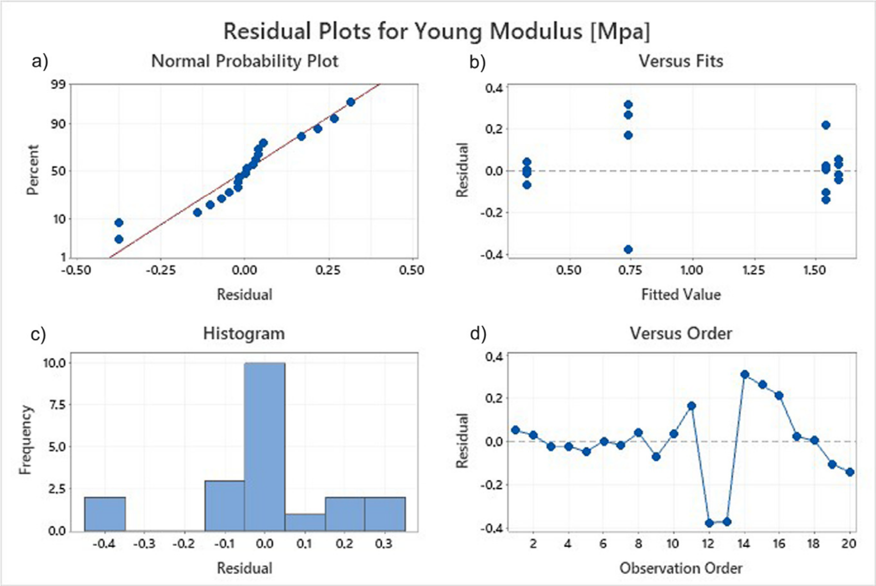

Figure S7. Analysis of residuals for Young modulus model in medium ingredients concentrations. (**a**) Homoscedasticity analysis. (**b**) Residuals analysis. (**c**) Residuals distributions. (**d**) Randomized analysis.

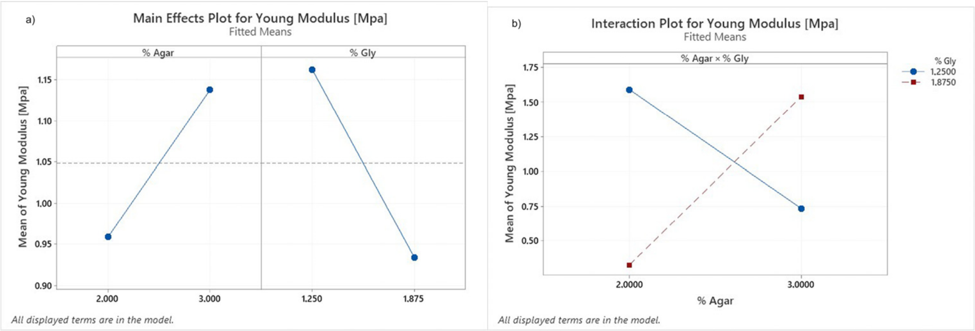

Figure S8. Effect analysis for Young modulus model in medium ingredients concentrations. (**a**) Main effects. (**b**) Interaction effect.

In Equation 5 the elongation at break model in medium ingredients concentrations

$$\text{Elongation at break [\%]} = 35.6 - (0.29 \times \%\text{Agar}) + (7.33 \times \%\text{Gly}) - (1.12 \times \%\text{Agar} \times \%\text{Gly}) \quad (S5)$$

Table S17. Statistic parameters for elongation at break model in medium ingredients concentrations

Term	Effect	Coef	SE Coef	T-Value	P-Value	VIF
Constant		41.981	0.554	75.74	0.000	
% Agar	-2.042	-1.021	0.554	-1.84	0.084	1.00
% Gly	2.831	1.415	0.554	2.55	0.021	1.00
% Agar × % Gly	-0.350	-0.175	0.554	-0.32	0.756	1.00

Table S18. The correlation coefficient of the elongation at break model in medium ingredients concentrations.

S	R-sq	R-sq(adj)	R-sq(pred)
2.47884	38.50%	26.96%	3.90%

Table S19. Analysis of variance of the elongation at break model in medium ingredients concentrations.

Source	DF	Adj SS	Adj MS	F-Value	P-Value
Model	3	61.534	20.5112	3.34	0.046
Linear	2	60.920	30.4602	4.96	0.021
% Agar	1	20.848	20.8478	3.39	0.084
% Gly	1	40.073	40.0725	6.52	0.021
2-Way Interactions	1	0.613	0.6133	0.10	0.756
% Agar × % Gly	1	0.613	0.6133	0.10	0.756
Error	16	98.314	6.1446		
Total	19	159.848			

Table S20. Fits and Diagnostics for Unusual Observations for elongation at break model in medium ingredients concentrations.

Obs	Elongation at break [Mpa]	Fit	Resid	Std Resid
11	44.95	39.72	5.23	2.36
15	34.74	39.72	-4.98	-2.25

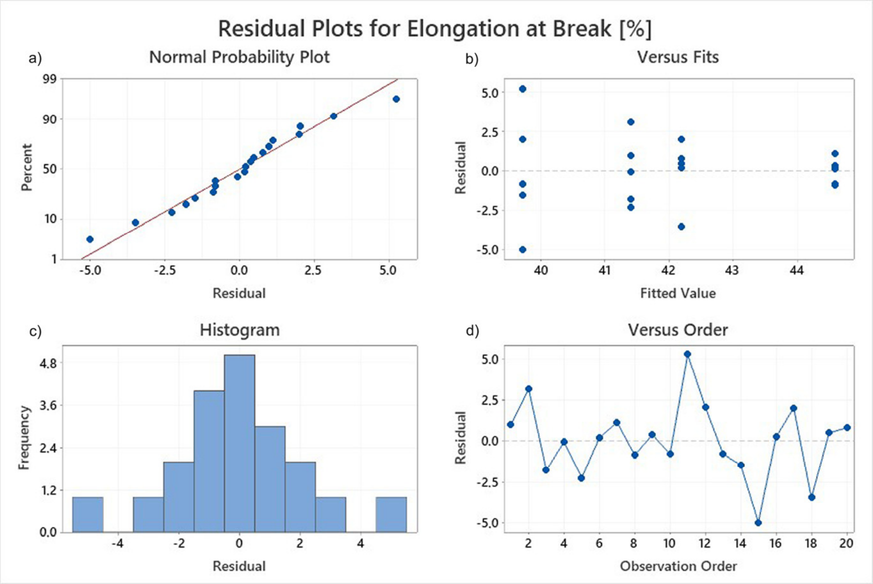

Figure S9. Analysis of residuals for elongation at break model in medium ingredients concentrations. (**a**) Homoscedasticity analysis. (**b**) Residuals analysis. (**c**) Residuals distributions. (**d**) Randomized analysis.

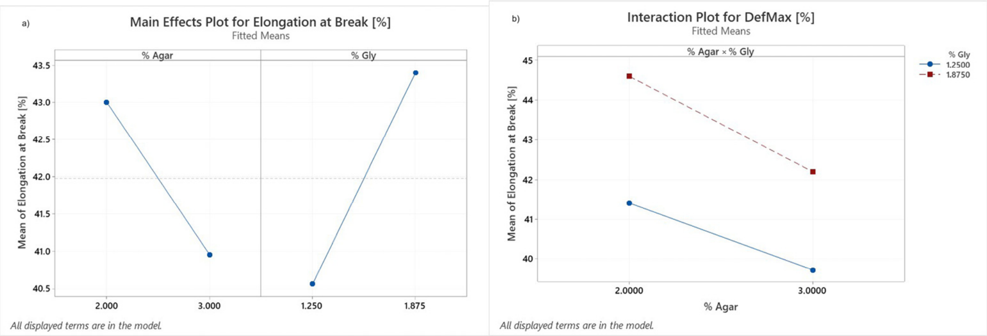

Figure S10. Effect analysis for elongation at break model in medium ingredients concentrations. (**a**) Main effects. (**b**) Interaction effect.

In Equation (6) the Ultimate Tensile Strenght (UTS) model in medium ingredients concentrations

$$\text{UTS [Mpa]} = 75.98 - (26.10 \times \%\text{Agar}) - (45.78 \times \%\text{Gly}) + (17.735 \times \%\text{Agar} \times \%\text{Gly}) \quad \text{(S6)}$$

Table S21. Statistic parameters for UTS model in medium ingredients concentrations

Term	Effect	Coef	SE Coef	T-Value	P-Value	VIF
Constant		8.476	0.123	69.05	0.000	
% Agar	1.612	0.806	0.123	6.57	0.000	1.00
% Gly	-0.901	-0.451	0.123	-3.67	0.002	1.00
% Agar × % Gly	5.542	2.771	0.123	22.58	0.000	1.00

Table S22. The correlation coefficient of the UTS model in medium ingredients concentrations.

S	R-sq	R-sq(adj)	R-sq(pred)
0.548936	97.25%	96.74%	95.71%

Table S23. Analysis of variance of the UTS model in medium ingredients concentrations.

Source	DF	Adj SS	Adj MS	F-Value	P-Value
Model	3	170.636	56.879	188.76	0.000
Linear	2	17.050	8.525	28.29	0.000
% Agar	1	12.987	12.987	43.10	0.000
% Gly	1	4.063	4.063	13.48	0.002
2-Way Interactions	1	153.585	153.585	509.69	0.000
% Agar × % Gly	1	153.585	153.585	509.69	0.000
Error	16	4.821	0.301		
Total	19	175.457			

Table S24. Fits and Diagnostics for Unusual Observations for UTS model in medium ingredients concentrations.

Obs	UTS [Mpa]	Fit	Resid	Std Resid
5	9.824	10.892	-1.068	-2.17
12	5.851	6.961	-1.110	-2.26

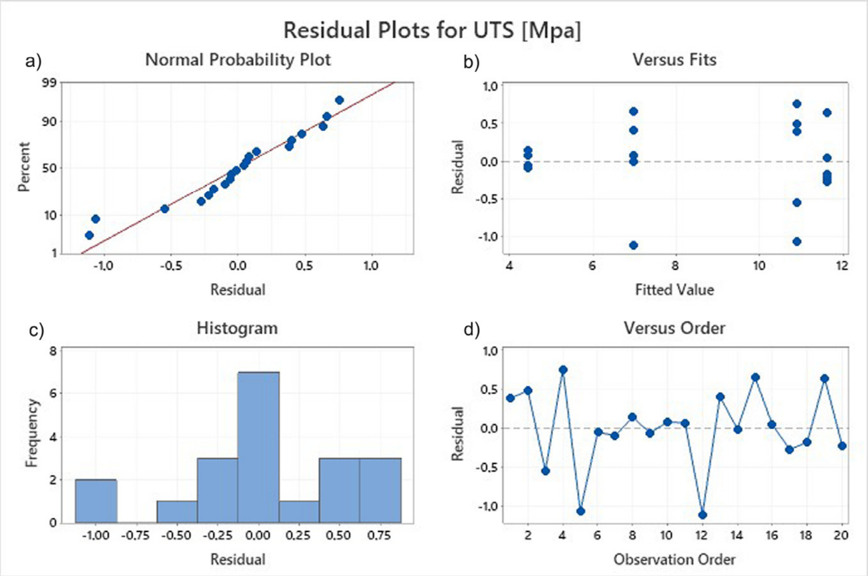

Figure S11. Analysis of residuals for UTS model in medium ingredients concentrations. (**a**) Homoscedasticity analysis. (**b**) Residuals analysis. (**c**) Residuals distributions. (**d**) Randomized analysis.

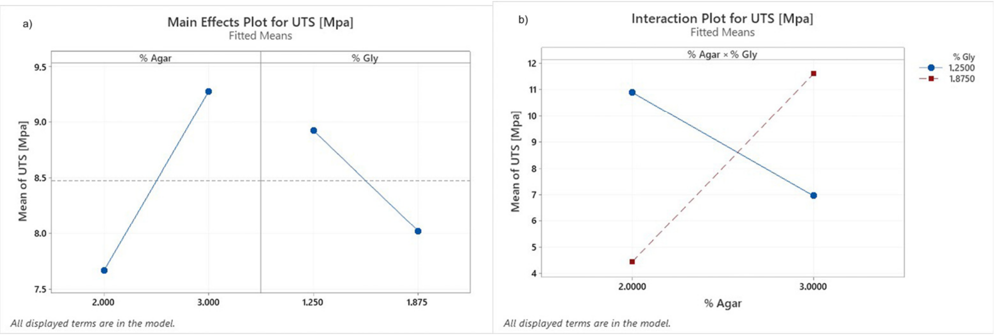

Figure S12. Effect analysis for UTS model in medium ingredients concentrations. (**a**) Main effects. (**b**) Interaction effect.

3. ANOVA - Data range 3: High ingredients concentrations:

In Equation 7 the Young modulus model in high ingredients concentrations

$$\text{Young Modulus [Mpa]} = 1.457 - (0.2275 \times \%\text{Agar}) - (0.1981 \times \%\text{Gly}) + (0.0387 \times \%\text{Agar} \times \%\text{Gly}) \quad \text{(S7)}$$

Table S25. Statistic parameters for Young modulus model in high ingredients concentrations

Term	Effect	Coef	SE Coef	T-Value	P-Value	VIF
Constant		0.3641	0.0660	5.51	0.000	
% Agar	0.3314	0.1657	0.0660	2.51	0.026	1.13
% Gly	0.1816	0.0908	0.0660	1.37	0.193	1.13
% Agar × % Gly	0.4139	0.2069	0.0660	3.13	0.008	1.13

Table S26. The correlation coefficient of the Young modulus model in high ingredients concentrations.

S	R-sq	R-sq(adj)	R-sq(pred)
0.251904	61.00%	52.00%	38.94%

Table S27. Analysis of variance of the Young modulus model in high ingredients concentrations.

Source	DF	Adj SS	Adj MS	F-Value	P-Value
Model	3	1.2900	0.43001	6.78	0.005
Linear	2	0.6900	0.34499	5.44	0.019
% Agar	1	0.3995	0.39945	6.29	0.026
% Gly	1	0.1199	0.11986	1.89	0.193
2-Way Interactions	1	0.6228	0.62283	9.82	0.008
% Agar × % Gly	1	0.6228	0.62283	9.82	0.008
Error	13	0.8249	0.06346		
Total	16	2.1150			

Table S28. Fits and Diagnostics for Unusual Observations for Young modulus model in high ingredients concentrations.

Obs	Young Modulus [Mpa]	Fit	Resid	Std Resid
1	0.997	0.315	0.683	3.03

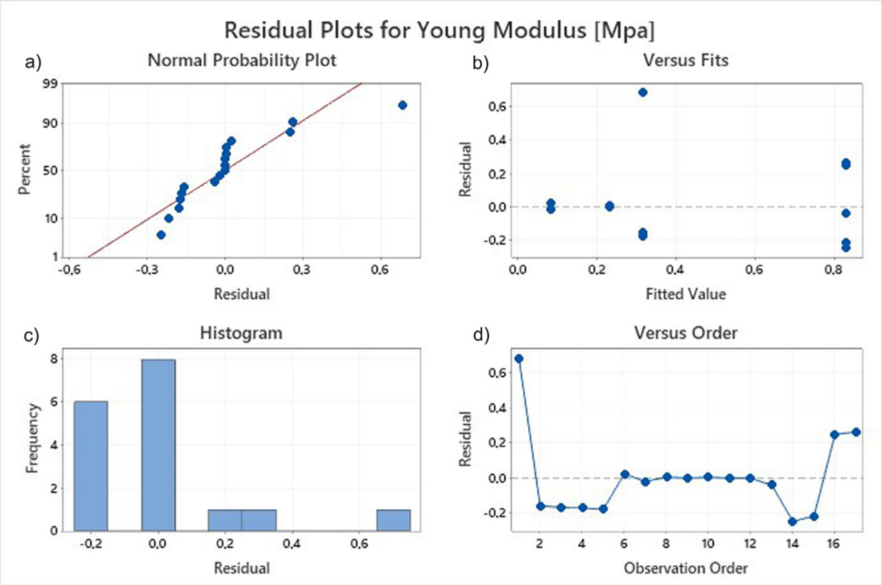

Figure S13. Analysis of residuals for Young modulus model in high ingredients concentrations. (**a**) Homoscedasticity analysis. (**b**) Residuals analysis. (**c**) Residuals distributions. (**d**) Randomized analysis.

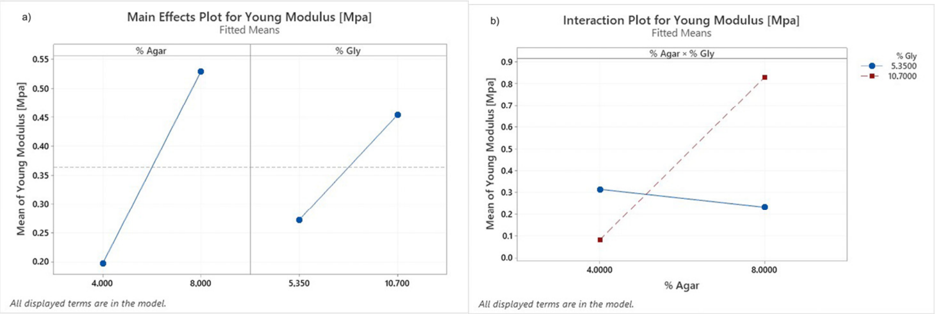

Figure S14. Effect analysis for Young modulus model in high ingredients concentrations. (**a**) Main effects. (**b**) Interaction effect.

In Equation 8 the elongation at break model in high ingredients concentrations

$$\text{Elongation at break [\%]} = -70.0 + (12.59 \times \%\text{Agar}) + (23.11 \times \%\text{Gly}) - (2.612 \times \%\text{Agar} \times \%\text{Gly}) \quad \text{(S8)}$$

Table S29. Statistic parameters for elongation at break model in high ingredients concentrations

Term	Effect	Coef	SE Coef	T-Value	P-Value	VIF
Constant		65.18	1.72	38.00	0.000	
% Agar	-33.49	-16.75	1.72	-9.76	0.000	1.13
% Gly	39.80	19.90	1.72	11.60	0.000	1.13
% Agar × % Gly	-27.95	-13.97	1.72	-8.15	0.000	1.13

Table S30. The correlation coefficient of the elongation at break model in high ingredients concentrations.

S	R-sq	R-sq(adj)	R-sq(pred)
6.54284	93.78%	92.34%	85.85%

Table S31. Analysis of variance of the elongation at break model in high ingredients concentrations.

Source	DF	Adj SS	Adj MS	F-Value	P-Value
Model	3	8390.0	2796.67	65.33	0.000
Linear	2	7773.0	3886.48	90.79	0.000
% Agar	1	4079.2	4079.21	95.29	0.000
% Gly	1	5759.4	5759.44	134.54	0.000
2-Way Interactions	1	2840.1	2840.06	66.34	0.000
% Agar × % Gly	1	2840.1	2840.06	66.34	0.000
Error	13	556.5	42.81		
Total	16	8946.5			

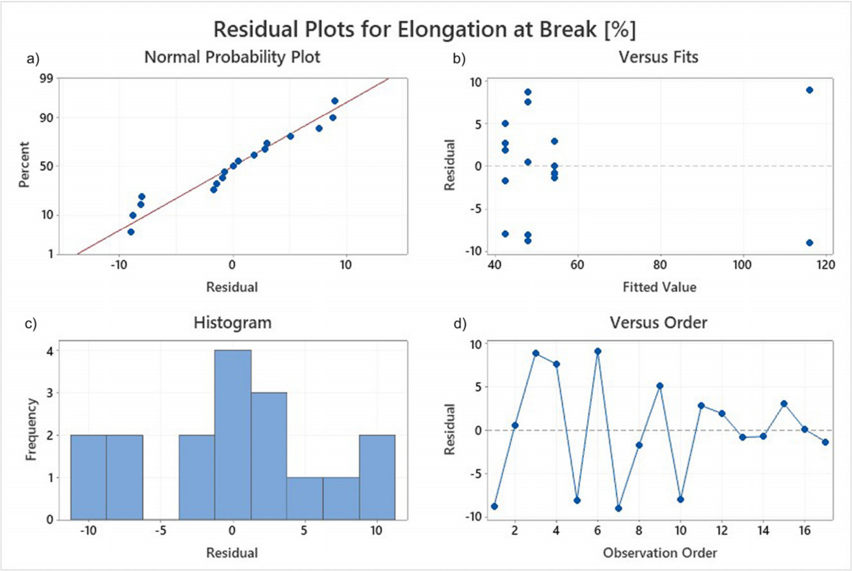

Figure S15. Analysis of residuals for elongation at break model in high ingredients concentrations. (**a**) Homoscedasticity analysis. (**b**) Residuals analysis. (**c**) Residuals distributions. (**d**) Randomized analysis.

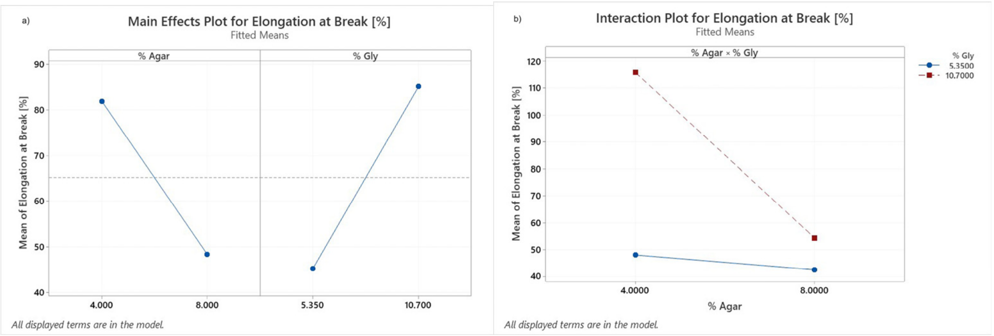

Figure S16. Effect analysis for elongation at break model in high ingredients concentrations. (**a**) Main effects. (**b**) Interaction effect.

In Equation 9 the Ultimate Tensile Strenght (UTS) model in high ingredients concentrations

$$UTS\ [Mpa] = 9.18 - (1.240 \times \%Agar) - (1.261 \times \%Gly) + (0.2801 \times \%Agar \times \%Gly) \quad (S9)$$

Table S32. Statistic parameters for UTS model in high ingredients concentrations

Term	Effect	Coef	SE Coef	T-Value	P-Value	VIF
Constant		5.104	0.160	31.99	0.000	
% Agar	4.031	2.015	0.160	12.63	0.000	1.13
% Gly	2.245	1.123	0.160	7.04	0.000	1.13
% Agar × % Gly	2.997	1.499	0.160	9.39	0.000	1.13

Table S33. The correlation coefficient of the UTS model in high ingredients concentrations.

S	R-sq	R-sq(adj)	R-sq(pred)
0.608422	96.46%	95.64%	94.10%

Table S34. Analysis of variance of the UTS model in high ingredients concentrations.

Source	DF	Adj SS	Adj MS	F-Value	P-Value
Model	3	131.185	43.7285	118.13	0.000
Linear	2	103.034	51.5171	139.17	0.000
% Agar	1	59.083	59.0829	159.61	0.000
% Gly	1	18.335	18.3350	49.53	0.000
2-Way Interactions	1	32.668	32.6683	88.25	0.000
% Agar × % Gly	1	32.668	32.6683	88.25	0.000
Error	13	4.812	0.3702		
Total	16	135.998			

Table S35. Fits and Diagnostics for Unusual Observations for UTS model in high ingredients concentrations.

Obs	UTS [Mpa]	Fit	Resid	Std Resid
3	4.562	3.465	1.097	2.02

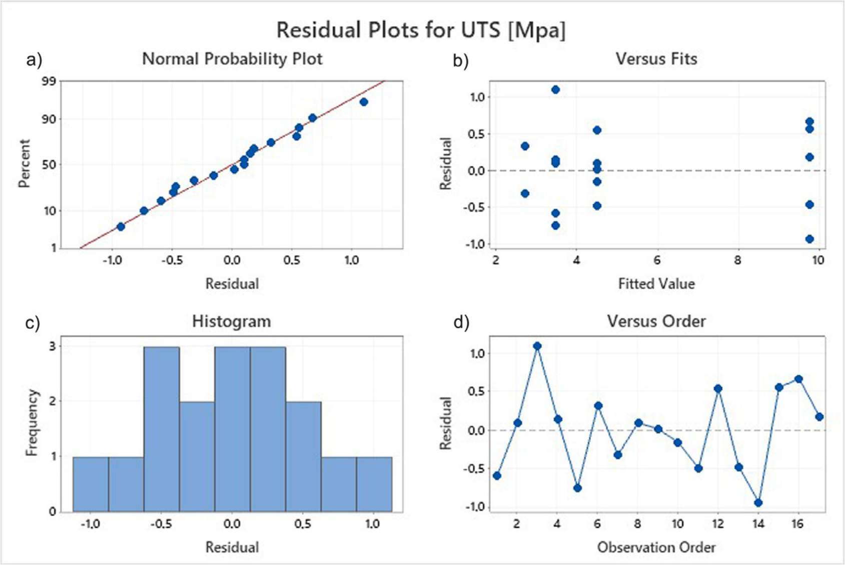

Figure S17. Analysis of residuals for UTS model in high ingredients concentrations. (**a**) Homoscedasticity analysis. (**b**) Residuals analysis. (**c**) Residuals distributions. (**d**) Randomized analysis.

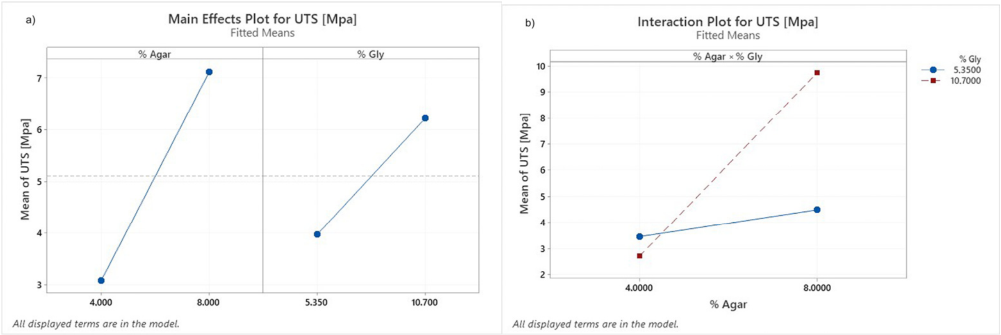

Figure S18. Effect analysis for UTS model in high ingredients concentrations. (**a**) Main effects. (**b**) Interaction effect.
